# Modern Aspects of Inflammation

**Published:** 1893-12-16

**Authors:** 


### MODERN ASPECTS OF INFLAMMATION.

Inflammation of some sort or another plays a prominent part in so many of the serious illnesses of mankind that no apology is necessary for introducing to our readers the modern views concerning it, especially as an excellent translation of Metchnikoff's "Comparative Pathology of Inflammation" has just been published by Messrs. Kegan Paul, and therefore it is now easy for each reader to expand for himself the short summary we propose to give, and we strongly advise them to do so. The essential phenomenon of inflammation is the endeavour on the part of the protoplasm of the affected organism to, in some way, get rid of a harmful object. Generally this is effected by the cells of the affected organism digesting the irritant. In the case of protozoa when they are invaded by a harmful object, often a bacillus, the whole organism takes part in this digestive process, while from sponges upwards this fighting the foreign harmful object is confined to special cells, derived from the mesoderm, and these warrior cells, which usually, if victorious, eat their victim, are called phagocytes. In the lower animals that have no vascular system, when the injurious agent is introduced into the body the phagocytes are usually attracted to it, and a number of them assembled together, surround, and proceed to destroy, usually by digestion, the invader or invaders. When in such a case as this the phagocytes are attracted to the invader, there is supposed to be some attractive power between the invader and them, by which they move towards it; this attractive power is called a positive chemiotactic action; the phenomenon is called positive chemiotaxis. In some cases, especially of unicellular animals, the harmful agent is avoided by the animal (or if not unicellular, by the phagocytes) retreating from the invader; in such a case the phenomenon is called one of negative chemiotaxis.

Now the phagocytes of every animal throughout the whole animal kingdom have these chemiotactic properties, and this chemiotactic phagocytesis is the only phenomenon of inflammation which is constantly met with in all animals, and therefore in those creatures which are so low in the animal scale as to be without a vascular system, it is the only inflammatory phenomenon present. We must consequently regard it as the fundamental fact in inflammation.

Passing now to the consideration of animals with a vascular system we notice that in the most lowly the only value of the vessels in inflammation is that they form a means of rapidly transmitting the phagocytes to the seat of war. They are as passive as military roads, but this is only the case with a very lowly organised vascular system in communication with the general body cavity, for we soon meet with the phenomenon of diapedesis which, as is well known, consists in the passage of white blood corpuscles, and especially such of them as are phagocytes, through the vessel walls. This is because the endothelial cells of the vessels are endowed with the property of independent movement, in virtue of which they can so alter their shape as to leave spaces between adjacent endothelial cells through which phagocytes can pass. When the irritant foreign body is present these endothelial cells, by allowing the phagocytes to pass between them, thus co-operate with them to destroy the invader, for the phagocytes, brought to the seat of war, proceed to attempt this directly they have passed through the vessel wall. If they succeed the organism recovers; if they fail, and the invading foreign agent is a bacillus which is constantly forming a virulent toxine, the organism dies.

Probably in all cold-blooded animals the inflammatory phenomena do not advance beyond this, but in warm-blooded animals the nervous system participates, with the result that the irritant reflexly causes vascular dilatation in its neighbourhood. The advantage of this is obvious, for by its means more blood, and consequently a great many phagocytes, are carried to the inflamed area. This vascular dilatation explains the heat and redness of the inflamed area. The cause of the swelling is also to a large extent the vascular dilatation, but at present we hardly understand why fluid should transude into the tissues around the dilated blood vessels. The rise of temperature in inflammation is probably due to the toxines developed at the seat of injury.

It follows from what we have just said that all the phenomena of inflammation, except the phagocytosis and the rise of temperature, are due to the fact that the harmful agent is outside the blood vessels. It is therefore very interesting to take a disease in which the invader is inside them. Such diseases are relapsing fever, in which the spirillum is in the blood itself, as also is the plasmodium malariæ in ague. In both these diseases the fight between the invader and the phagocytes takes place in the blood, and the toxines produced cause a great rise of temperature. We have here a case of inflammation unaccompanied by diapedesis; it is really an instance of inflammation in the blood itself, in fact, a "hemitis," as Piorry considered it might be many years ago.

We have mentioned the active co-operation of the endothelial cells of the vessels, which help by allowing the intravascular leucocytes to pass between them. These endothelial cells have other properties. In the first place they are genuine phagocytes, for often they may be seen to have ingested and to be digesting bacilli, such as those of anthrax, and in the next place they often exhibit marked positive chemiotaxis, for when an irritant is introduced they lengthen out towards it, and thus new vascular loops are formed, as has been for many years known to be of frequent occurrence in inflammation, and consequently blood containing many phagocytes is better conveyed to the seat of war.

Hitherto we have spoken as if the only means that the phagocytes possessed of killing invaders was by digesting them or avoiding them, but they have another. A study of cases of tuberculosis which have caseated, and in which lime salts have been deposited to form cretaceous masses such as are often met with in the lungs of those who have recovered from pulmonary tuberculosis, shows that in order to defend itself from the attacks of the phagocytes the bacillus secretes layer after layer of thick material around itself, but the phagocytes reply by secreting lime salts, which ultimately wall in the bacillus with a cretaceous wall, and as a result of this it finally dies.

To sum up, it is thus seen that Metchnikoff's biological or comparative theory of inflammation is founded on a comparative study of pathological phenomena, and that the essential phenomena of inflammation represent an actual struggle between the phagocytes and the irritant agent. It must indeed be regarded as a phagocytic reaction on the part of the organism carried out by the mobile phagocytes, sometimes alone and sometimes with the aid of the vascular endothelial phagocytes and of the nervous system.

